# The Preparation of High-Performance MoO_3_ Nanorods for 2.1 V Aqueous Asymmetric Supercapacitor

**DOI:** 10.3390/nano14242029

**Published:** 2024-12-17

**Authors:** Ziyu Lian, Xiling Mao, Yi Song, Kaihua Yao, Ruifeng Zhang, Xinyu Yan, Mengwei Li

**Affiliations:** School of Instrument and Electronics, North University of China, Taiyuan 030051, China; sz202206017@st.nuc.edu.cn (Z.L.); sz202306146@st.nuc.edu.cn (Y.S.); b20220641@st.nuc.edu.cn (K.Y.); s202406091@st.nuc.edu.cn (R.Z.); 20200149@nuc.edu.cn (X.Y.)

**Keywords:** aqueous electrolyte, MoO_3_, wide voltage window, supercapacitor, hydrothermal method

## Abstract

In order to broaden the working voltage (1.23 V) of aqueous supercapacitors, a high-performance asymmetric supercapacitor with a working voltage window reaching up to 2.1 V is assembled using a nanorod-shaped molybdenum trioxide (MoO_3_) negative electrode and an activated carbon (AC) positive electrode, as well as a sodium sulfate–ethylene glycol ((Na_2_SO_4_-EG) electrolyte. MoO_3_ electrode materials are fabricated by adjusting the hydrothermal temperature, hydrothermal time and solution’s pH value. The specific capacity of the optimal MoO_3_ electrode material can reach as high as 244.35 F g^−1^ at a current density of 0.5 A g^−1^. For the assembled MoO_3_//AC asymmetric supercapacitor with a voltage window of 2.1 V, its specific capacity, the energy density, and the power density are 13.52 F g^−1^, 8.28 Wh kg^−1^, and 382.15 W kg^−1^ at 0.5 A g^−1^, respectively. Moreover, after 5000 charge–discharge cycles, the capacity retention rate of the device still reaches 109.2%. This is mainly attributed to the small particle size of MoO_3_ nanorods, which can expose more electrochemically active sites, thus greatly facilitating the transport of electrolyte ions, immersion at the electrolyte/electrolyte interface and the occurrence of electrochemical reactions.

## 1. Introduction

With the rapid development of the social economy, limited fossil fuel resources and the ever-increasing population have confronted mankind with energy issues. Supercapacitors (electrochemical capacitors) have drawn widespread attention due to advantages such as their high power density and long cycle life [1,2,3,4,5], making them highly promising candidates for the next-generation high-efficiency energy storage devices used in fields such as electronic devices, power quality systems, hybrid electric vehicles, and smart grids [6]. Compared with rechargeable batteries, the relatively low energy density of supercapacitors has seriously hindered their further commercial application. Therefore, a great deal of time has been devoted to researching how the energy density of supercapacitors can be improved while retaining their high power density and long cycle life. According to the energy density equation (E = CV^2^/2) [7], the energy density (E) of supercapacitors can be enhanced by either expanding the working voltage (V) or increasing the specific capacitance (C).

Supercapacitor electrolytes can effectively improve the working voltage (V). Electrolytes can be classified into non-aqueous electrolytes and aqueous electrolytes. In particular, non-aqueous electrolytes have become an attractive candidate in current applications due to their relatively wide working voltage, long cycle life, and high power density. However, they also have problems such as a high cost, relatively low ionic conductivity, and low safety. In recent years, aqueous electrolytes have attracted attention and been widely studied because of their advantages, which include a low cost, relatively high ionic conductivity, and high levels of safety [8,9]. Nevertheless, their low electrochemical stability window (1.23 V) has limited their practical application [10]. In terms of this issue, Alessio D’Alessandro and others have expanded the voltage window to 1.4 V by adding amino acids into the electrolyte [11]. Wang et al. designed and studied a cyano-substituted diquinoxaline phenazine (3CN-DPZ) organic electrode for aqueous alkaline ion batteries with a voltage of 2.0 V [12]. Liu et al. investigated 14M potassium formate and 6.4 m and 21 m cesium acetate, and achieved a high energy density and a battery voltage of up to 1.7 V [13].

Transition metal oxides usually have multiple oxidation states and can undergo reversible redox reactions, thereby providing a relatively high specific capacitance [14]. For example, manganese dioxide (MnO_2_ [15]), nickel oxide (NiO [16]), ferric oxide (Fe_2_O_3_ [17]), cobalt oxide (Co_2_O_3_ [18]), molybdenum trioxide (MoO_3_ [19]), etc., can store a large amount of charge through Faradaic pseudo-capacitance reactions within a certain potential range. This can promote the transmission of electrons in the electrode materials, reduce the internal resistance, and improve the power performance of supercapacitors. In addition, transition metal oxides usually possess good chemical and thermal stabilities. They can maintain the integrity of their structures during the charging and discharging processes, thus prolonging the service life of supercapacitors. Molybdenum, as a typical transition metal oxide, features multiple valence states and high electrochemical activity. Compounds of molybdenum, as electrochemical energy storage materials, have been widely researched and applied in both supercapacitor and lithium-ion battery materials.

As a transition metal oxide, MoO_3_ has a relatively high theoretical specific capacitance due to the multi-electron transfer process during the proton insertion–desorption process; it has thus attracted widespread attention as a pseudo-capacitor material applied to energy storage [20,21,22,23]. Additionally, the cathode needs to receive electrons at a lower potential for the reduction reaction during the charging/discharging process, and the redox potential of MoO_3_ is relatively low; therefore, the redox reaction of MoO_3_ can occur stably in this lower potential, resulting in more storage and the release of charge. The conversion process in the polyvalent state of MoO_3_ can result in the transfer of multiple electrons, so that the unit mass of MoO_3_ can store a relatively large amount of charge, thus providing a high theoretical capacity for the supercapacitor (1170 mAh g^−1^) [24,25]. Therefore, nanorod-shaped MoO_3_ can be selected as a cathode in this paper. Compared with crystalline MoO_3_, nanoscale MoO_3_ is highly favored because of its ordered structure, good chemical stability, high elasticity, low internal energy, and large surface area, enabling it to achieve isotropy [26].

Therefore, nanorod-shaped MoO_3_ was successfully prepared by a simple one-step hydrothermal method in this work. The optimal MoO_3_ material was obtained by adjusting the experimental conditions (hydrothermal time, hydrothermal temperature, and pH value). This nanostructure enables complete contact between the electrolyte and the active material, resulting in rapid reaction kinetics. The material prepared under the optimal conditions (180 °C/24 h/pH = 0.36) can reach a specific capacity of 244.35 F g^−1^ at a current density of 0.5 A g^−1^. In addition, an AC//MoO_3_ asymmetric supercapacitor was assembled with a Na_2_SO_4_-EG electrolyte, in which an aqueous electrolyte with a low salt concentration (1 M Na_2_SO_4_(mol kg⁻^1^)) was used, and the ethylene glycol (EG) was used as an additive. In this assembled device, due to the strong coordination between EG molecules and Na^+^ ions, some H_2_O molecules are not in the Na^+^ solvation shell and do not participate in the decomposition process on the electrode surface, thus achieving a wide electrochemical window (2.1 V) and demonstrating its application prospects in the field of energy storage devices.

## 2. Materials and Methods

### 2.1. Preparation

Analytically pure (99%) ammonium molybdate ((NH_4_)_2_MoO_4_) produced by Shanghai Macklin Biochemical Co., Ltd. (Shanghai, China); superfine-grade (65–68%) concentrated nitric acid produced by Shanghai Aladdin Biochemical Technology Co., Ltd. (Shanghai, China); analytically pure (99.7%) anhydrous ethanol produced by Sinopharm Chemical Reagent Co., Ltd. (Shanghai, China); analytically pure (99%) ethylene glycol produced by Shanghai Macklin Biochemical Co., Ltd. (Shanghai, China); polyvinylidene fluoride (PVDF) produced by Shanghai Aladdin Biochemical Technology Co., Ltd (Shanghai, China).; and capacitor-grade AC produced by Shanghai Aladdin Biochemical Technology Co., Ltd. (Shanghai, China) were obtained.

**Figure 1:**
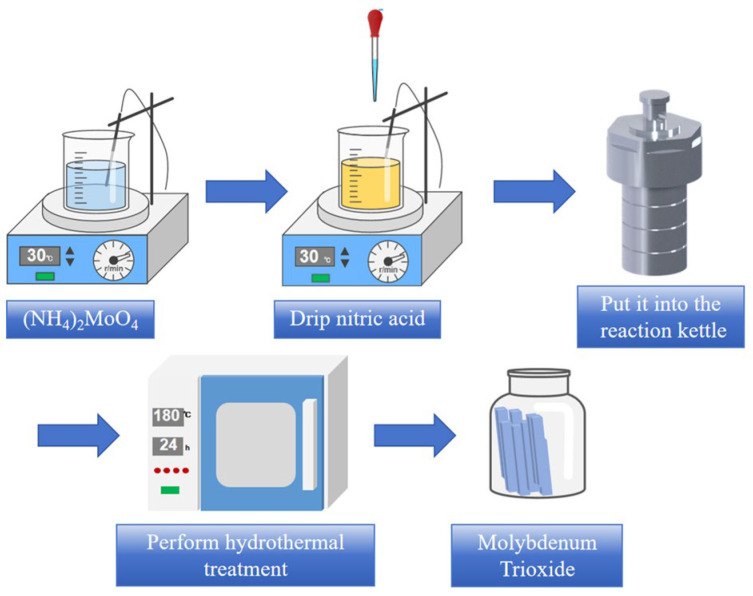
Schematic diagram of preparation of MoO_3_.

Preparation of MoO_3_ sample: The process of preparing MoO_3_ materials is shown in Figure 1. Firstly, 196 mg of (NH_4_)_2_MoO_4_ and 76 mL of pure water were put into a beaker subjected to magnetic stirring for one hour for later use. Then, a moderate amount (2.6 mL, 1.7 mL and 0.8 mL) of nitric acid (HNO_3_, 68%) was poured into the aforesaid solution to adjust the pH value (0.36, 0.58 or 0.84). Additionally, the hydrothermal time (12 h, 18 h, 24 h and 30 h) and hydrothermal temperature (160 °C, 180 °C and 200 °C) were used as the experimental variables for the hydrothermal reaction to obtain the optimal MoO_3_ sample; this was followed by centrifugation and freeze drying.

Preparation of electrode: Firstly, the 10 mg mL^−1^ ethanol–PVDF solution was prepared, and the prepared MoO₃ or activated carbon (AC) were dried and put on standby. Then, the MoO_3_ (AC), conductive carbon black and PVDF binder were mixed uniformly at a ratio of 8:1:1, and the electrode (MoO_3_ or AC) was obtained on a carbon cloth substrate; this was followed by drying at 70 °C for 12 h.

Preparation of the electrolyte: Firstly, 14.2 g of sodium sulfate powder (Na_2_SO_4_) and 100 mL of purified water were poured into the beaker and stirred overnight to obtain the transparent Na_2_SO_4_ electrolyte; then, 30 mL of ethylene glycol (EG) and 70 mL of the aforementioned Na_2_SO_4_ solution were mixed uniformly to prepare the Na_2_SO_4_-EG electrolyte.

Assembly of the supercapacitor: The MoO₃//AC asymmetric supercapacitor was assembled in an electrolytic cell by the MoO₃ negative electrode, AC positive electrode, and Na_2_SO_4_-EG electrolyte. In contrast, the MoO₃//AC asymmetric device with an Na_2_SO_4_ electrolyte was also fabricated to further explore the charge storage process of an asymmetric device with a Na_2_SO_4_-EG electrolyte.

### 2.2. Characterization

A field emission scanning electron microscope (SEM, Zeiss Merlin Compact from Jena, Germany) and X-ray energy-dispersive spectroscopy (EDS) were employed to characterize the microscopic morphology and elemental distribution of the samples. X-ray diffraction (XRD, PRO MPD from Utrecht, The Netherlands) was utilized to analyze the microscopic structure of the substances. A Fourier transform infrared (FT-IR) spectrometer (ThermoScientific NicoletiS20, Waltham, MA, USA) was used to analyze the molecular structure and information about the chemical bonds (functional groups) of the samples based on the position and intensity of the specific absorption peaks of chemical bonds or groups within the samples. The specific surface area and porosity of the electrode materials were characterized by employing the fully automatic specific surface area and porosity analyzer (Micromeritics ASAP 2460, Norcross, GA, USA).

### 2.3. Electrochemical Measurements

The electrochemical tests of the electrode materials were completed on the CHI760E electrochemical workstation (Shanghai, China). A three-electrode testing system was adopted, with the prepared electrode sheet used as the working electrode, the platinum electrode used as the counter electrode, and the saturated calomel electrode used as the reference electrode. A 1 mol/L Na_2_SO_4_ solution was used as the electrolyte. All the electrochemical tests were carried out at room temperature. Cyclic voltammetry tests (CV), galvanostatic charge–discharge tests (GCD), and electrochemical impedance spectroscopy tests (EIS) were used to test and compare the electrode materials prepared at different hydrothermal temperatures (160 °C, 180 °C, 200 °C), different pH values, and different hydrothermal times (12 h, 18 h, 24 h, 30 h). The voltage window for CV and GCD was −1 V to −0.3 V. According to the GCD curve, the formula for calculating the specific capacity is as follows:

(1) $$C=\frac{I}{\frac{dV}{dt}}=\frac{I\int Vdt}{\int_{V_{1}}^{V_{2}}VdV}$$

In the formula, *C*, *I*, *V*, *V*_1_, *V*_2_ and t represent the specific capacitance (unit: F·g^−1^), applied current density (unit: A·g^−1^), working voltage window (unit: V), initial voltage (unit: V), cut-off voltage (unit: V) and discharge time (unit: s), respectively.

The formula for calculating the loading mass of the positive and negative electrodes of an asymmetric supercapacitor (ASC) is as follows [27]:

(2) $$\frac{m^{+}}{m^{-}}=\frac{C^{-}\Delta V^{-}}{C^{+}\Delta V^{+}}$$

In the formula, m^+^ and m^−^ are the loading masses of the positive and negative electrodes of the ASC (unit: g), respectively; *C^+^* and *C^−^* are the specific capacitances of the positive and negative electrode materials (unit: F·g^−1^), respectively; and Δ*V^+^* and Δ*V^−^* are the positive and negative voltage windows (unit: V), respectively. Therefore, the loading masses of the MoO_3_ and AC electrode materials were about 3.75 mg and 4.1 mg, respectively.

The energy density and power density of the supercapacitor battery are calculated using Equations (3) and (4), respectively:

(3) $$E=\frac{CV^{2}}{2}$$

(4) $$P=\frac{3600E}{t}$$

In the formula, *C* represents the specific capacitance of the battery (unit: F·g^−1^), *V* represents the size of the voltage window (unit: V), and *t* represents the discharge time (unit: h).

## 3. Results and Discussion

### 3.1. Structure and Morphology of Materials

**Figure 2:**
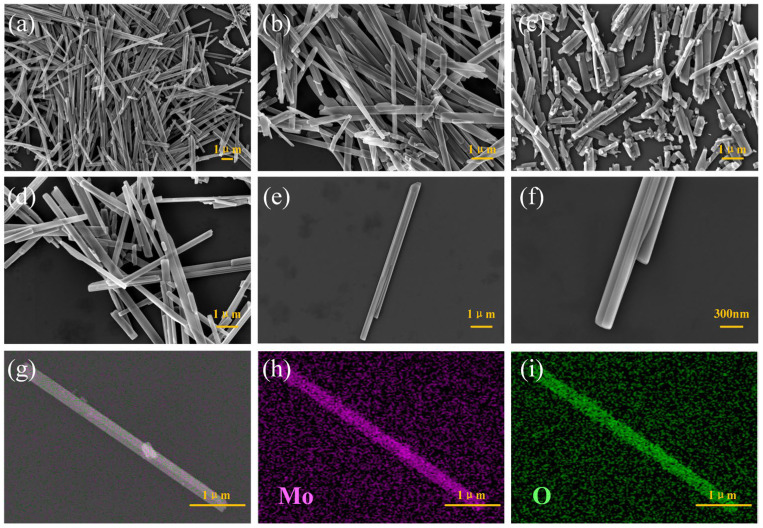
(**a**–**c**) SEM images of MoO_3_ at hydrothermal times of 12 h, 18 h, 30 h; (**d**–**f**) SEM image of MoO_3_ under hydrothermal time of 24 h; (**g**–**i**) EDS spectra of different element distributions in MoO_3_.

Figure 2a–d are the SEM images of MoO_3_ prepared under hydrothermal conditions for 12 h, 18 h, 30 h, and 24 h, respectively. It can be observed that the overall morphology shows a nanorod shape. The MoO_3_ nanorods prepared under the hydrothermal condition of 12 h are relatively long in length as a whole. With the increase in the hydrothermal time, when the hydrothermal time becomes 30 h, the nanorods are fractured. This is attributed to the fact that with the increase in the hydrothermal time, under a high temperature and high pressure, defects occur in the crystal structure of the material, and distortion or deformation takes place, which limits the growth of the length and consequently causes the fracture of the nanorods. Figure 2d–f are SEM images of multiple and single MoO_3_ nanorods under the hydrothermal time of 24 h. Compared with other hydrothermal conditions, the length of the nanorods under this condition is relatively moderate. The nanorod-shaped materials with a moderate length can achieve a good balance among properties such as dispersibility, anisotropy, and carrier transport. Neither the problems of agglomeration and carrier transport obstruction due to excessive length, nor the loss of the advantage of anisotropy because of the overly short length, will occur. Figure 2g–i are elemental mapping images (EDS). The figures prove the existence and uniform distribution of Mo and O elements, indicating that the MoO_3_ material has been successfully synthesized.

**Figure 3:**
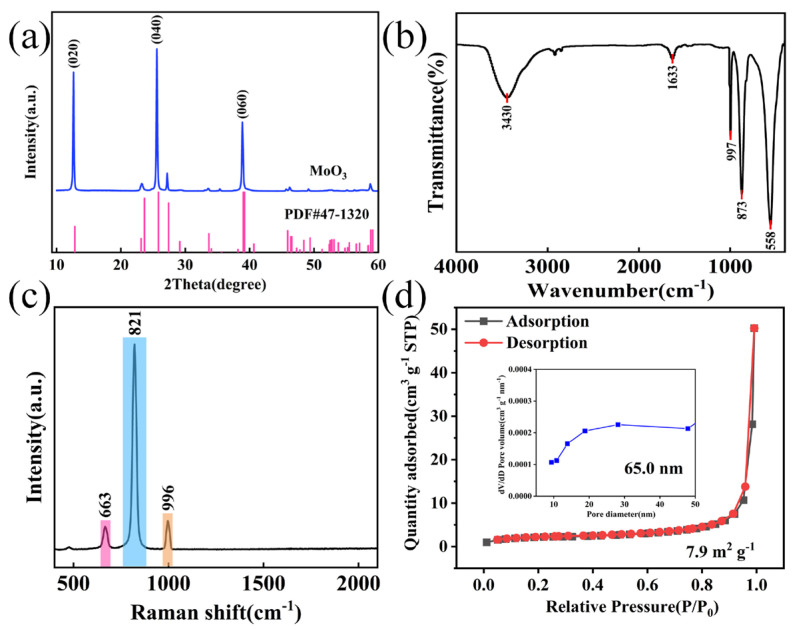
(**a**) XRD pattern of MoO_3_ material; (**b**) FT−IR spectrum of MoO_3_ material; (**c**) Raman spectrum of MoO_3_; (**d**) adsorption–desorption curves and pore size distribution diagrams.

To further explore the crystal structure of the prepared MoO_3_ materials, XRD tests were carried out on the materials. The results are shown in Figure 3a. Through comparison, it was found that the diffraction peaks of the samples were all consistent with the standard diffraction card JCPDF No. 47-1320, and no other impurity peaks appeared. This indicates that α-MoO_3_ was successfully prepared. Among them, the intensities of the diffraction peaks corresponding to the crystal planes (020), (040), and (060) were significantly higher than those of the diffraction peaks of other crystal planes, which indicates that the samples have good crystallinity. Figure 3b shows the FT-IR spectrum of MoO_3_. The peak at 997 cm^−1^ is caused by the stretching of the terminal oxygen of Mo = O. The peak at 873 cm^−1^ is attributed to the stretching of Mo-O-Mo. The peak at 558 cm^−1^ is due to the symmetric stretching of the O-Mo-O group. The characteristic peaks at 3430 cm^−1^ and 1633 cm^−1^ are caused by the stretching of the —OH group. The results indicate that the MoO_3_ material has been successfully synthesized [28]. Figure 3c presents the Raman spectrum of MoO_3_. It can be seen from the figure that three sharp Raman bands are displayed, located at 663 (B2g/B3g, vas, O—Mo—O stretch), 821 (Ag, vs, Mo = O stretching), and 996 cm^−1^ (Ag, vas, Mo = O stretching), respectively, all of which confirm the orthorhombic structure of α-MoO_3_ [29]. Figure 3d shows the BET and pore size distribution of the optimal MoO_3_ (180 °C/24 h/pH = 0.36). The curve inclines towards the Y-axis at the low-pressure (0–0.1) end, and there is an obvious adsorption hysteresis loop at the high-pressure (0.8–1) end, which belongs to a typical Type IV isotherm, indicating the existence of mesopores. The distribution of the corresponding pore size in the inset further demonstrates the presence of hierarchical micropores and mesopores.

Table 1 presents the comparison of BET for MoO_3_ prepared at different pH values. The specific surface area of the material prepared under the condition of pH = 0.36 is 7.9 m^2^ g^−1^, while the specific surface areas of the materials with pH = 0.58 and pH = 0.84 are 7.6 m^2^ g^−1^ and 7.5 m^2^ g^−1^, respectively. Since the increase in the BET surface area can provide more active sites and effective contact areas, it promotes the transfer and diffusion kinetics of the electrode surface and electrolyte components. According to the test, the BJH pore size under the condition of pH = 0.36 is larger (65 nm) than that for the other two conditions (48.7 nm, 40.0 nm).

### 3.2. Electrochemical Performance

**Figure 4:**
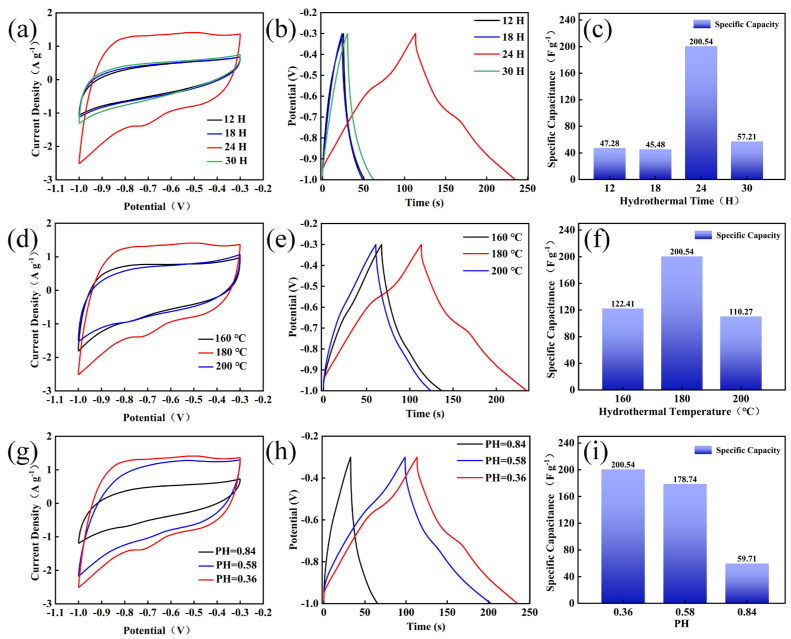
Comparison of electrochemical testing performance of MoO_3_ under different conditions: (**a**–**c**) comparison charts of CV (10 mV s^−1^), GCD (1 A g^−1^), and specific capacity (1 A g^−1^) under varying hydrothermal times; (**d**–**f**) comparison charts of CV (10 mV s^−1^), GCD (1 A g^−1^), and specific capacity (1 A g^−1^) at different hydrothermal temperatures; (**g**–**i**) comparison charts of CV (10 mV s^−1^), GCD (1 A g^−1^), and specific capacity (1 A g^−1^) under varying acidity indices.

Figure 4 shows the electrochemical performance of the electrode materials prepared under different conditions using a three-electrode system. Figure 4a−c present the electrochemical results of the samples under different hydrothermal times: 12 h, 18 h, 24 h and 30 h. Figure 4a shows the comparison of CV curves at a scanning rate of 10 mV s^−1^. It can be observed that when the hydrothermal time is 24 h, the integral area of the CV curve is the largest, indicating that it has the largest specific capacitance. Figure 4b shows the comparison of GCD curves at a current density of 1 A g^−1^. Moreover, when the hydrothermal time is 24 h, the charge–discharge time is the longest. The charge–discharge curves all present approximately symmetrical triangular shapes, indicating that the electrode material has good reversibility [30]. The specific capacitance calculated from the GCD is shown in Figure 4c, and the specific capacities of 12 h, 18 h, 24 h, and 30 h are 47.28, 45.48, 200.54, and 57.21 F g^−1^ at a current density of 1 A g^−1^, respectively. The corresponding energy densities are 3.22, 3.10, 13.65, and 3.89 Wh kg^−1^, and the corresponding power densities are 448.98, 440.42, 402.39 and 443.56 W kg^−1^. Therefore, it can be concluded that the sample with a hydrothermal time of 24 h possesses the best performance, which is consistent with the results of CV and GCD.

Figure 4d−f show the electrochemical tests under different hydrothermal temperatures (160 °C, 180 °C, 200 °C). These imply that when the hydrothermal temperature is 180 °C, the area of the CV curve is the largest, the charge–discharge time is the longest, and the performance is the best. Moreover, according to the GCD calculations, the specific capacitances of the electrode materials prepared at 160 °C, 180 °C, and 200 °C at 1 A g^−1^ are 122.41 F g^−1^, 200.54 F g^−1^, and 110.27 F g^−1^ respectively. The test results consistently show that MoO_3_ prepared at a hydrothermal temperature of 180 °C exhibits the best electrochemical performance. This is attributed to the fact that an excessively high temperature directly damages the structure of the material, while a temperature that is too low fails to meet the formation conditions of MoO_3_ nanorods.

Figure 4g−i present the electrochemical tests carried out on the samples prepared under a hydrothermal time of 24 h and a hydrothermal temperature of 180 °C, with pH being the variable. Specifically, 2.6 mL, 1.7 mL, and 0.8 mL of concentrated nitric acid were, respectively, used to prepare the samples with pH values of 0.36, 0.58 and 0.84. It can be seen from the CV diagrams that when the pH is 0.84, the integral area of the CV curve is the largest, indicating that its specific capacitance is also the largest. It can be seen from Figure 4h that as the acidity increases, the charge–discharge time also increases accordingly, and the electrochemical performance of the samples is gradually improved. Through the above comparative analysis, it can be concluded that under a hydrothermal time of 24 h, a hydrothermal temperature of 180 °C, and a pH of 0.36, the prepared MoO_3_ achieves the optimal electrochemical performance.

**Figure 5:**
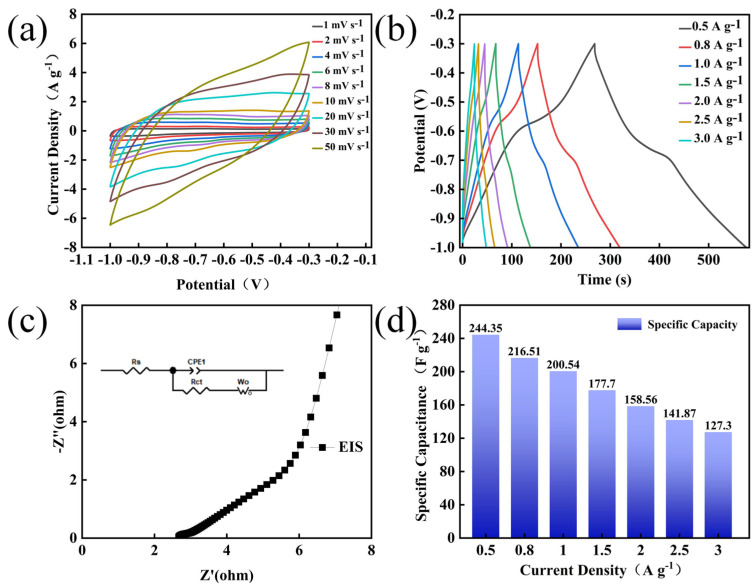
Electrochemical performance charts of MoO_3_ under hydrothermal conditions with a duration of 24 h, pH = 0.36, and at 180 °C: (**a**) CV diagram; (**b**) GCD diagram; (**c**) EIS diagram; (**d**) specific capacity diagram at 1 Ag^−1^.

To further evaluate the electrochemical performance of the prepared MoO_3_ nanomaterials, electrochemical tests were first carried out using a standard three-electrode system. Figure 5a shows the CV curves of the MoO_3_ nanocomposites at different scanning rates ranging from 1 to 50 mV s^−1^. It can be seen that with the increase in the scanning rate, all the curves exhibit similar shapes without obvious distortion, indicating that the CV curves at different scanning rates demonstrate good charge storage capabilities and efficient electrochemical responses at high rates [31]. Figure 5b presents the GCD curves with current densities ranging from 0.5 to 3 A g^−1^, and all the GCD curves display symmetrical charging and discharging processes without obvious plateaus, indicating good electrochemical reversibility. The EIS curve is shown in Figure 5c, where the inset is the simulated equivalent circuit diagram. In it, Rs and Rct are the series resistance and the constant phase element related to the double-layer capacitance and the Faraday resistance, respectively.

Figure 5d illustrates the comparative trend in the specific capacitance under different current densities. When the current density increases to five times the initial value, the specific capacitance retention rate is approximately 60%, demonstrating that the prepared electrode materials possess relatively good rate characteristics. The best performance was achieved under the experimental conditions of a hydrothermal time of 24 h, a hydrothermal temperature of 180 °C, and pH = 0.36. This is attributed to the nanorod-shaped MoO_3_, which allows the active sites to react more fully with ions, thereby improving the reaction rate and efficiency.

To further broaden the working voltage window of supercapacitors and improve their energy density, asymmetric supercapacitors were assembled using Na_2_SO_4_ and Na_2_SO_4_−EG as electrolytes, respectively; the charge storage mechanism of MoO_3_ can be calculated as follows [29]:

(5) $$MoO_{3}+Na^{+}+e^{-}\Leftrightarrow MoO_{3}Na$$

(6) $${\left(MoO_{3}\right)}_{surface}+Na^{+}+e^{+}\Leftrightarrow {\left(MoO_{3}Na^{+}\right)}_{surface}$$

**Figure 6:**
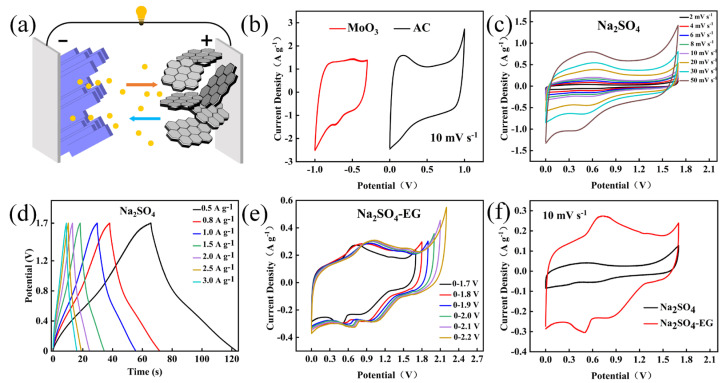
Electrochemical performance of MoO_3_//AC supercapacitors: (**a**) schematic diagram of the MoO_3_//AC supercapacitor electrolytic cell; (**b**) comparison chart of AC and MoO_3_ CV at 10 mV s^−1^; (**c**) Na_2_SO_4_ as electrolyte: CV plot at 0−1.7 V; (**d**) Na_2_SO_4_ as electrolyte: GCD plot at 0−1.7 V; (**e**) Na_2_SO_4_ −EG as electrolyte: CV plots at different voltage windows of 10 mV s^−1^; (**f**) comparison of CVs of Na_2_SO_4_ and Na_2_SO_4_ −EG at 10 mV/s within a voltage window of 0−1.7 V;.

Insights into the charge storage process involved in the surface adsorption and intercalation of Na^+^ ions on MoO_3_ electrode materials can be obtained using Equations (5) and (6). The excellent electrochemical performances of the MoO_3_//AC supercapacitor are ascribed to the synergistic effect between exposing more electrochemically active sites of MoO_3_ materials and the higher decomposition voltage of the Na_2_SO_4_-EG electrolyte compared with the electrochemical stability window (1.23 V) for commonly aqueous electrolyte. The results are shown in Figure 6. Figure 6a shows the schematic diagram of the structure of the asymmetric supercapacitor. Figure 6b presents the CV curves of the MoO_3_ and AC electrodes at a scanning rate of 10 mV s^−1^, respectively, indicating that, theoretically, the stable voltage window of the asymmetric supercapacitor can be extended to 1.7 V. First, an asymmetric supercapacitor was assembled with MoO_3_ as the negative electrode, AC as the positive electrode, and Na_2_SO_4_ as the electrolyte. Figure 6c shows the CV curve of the MoO_3_//AC tested under the voltage window of 0–1.7 V for this asymmetric supercapacitor. It can be seen that with the assembly of the asymmetric device, the CV curve is in good shape and the voltage window has been successfully broadened to 1.7 V. Figure 6d shows the GCD results of the device under different current densities. All the curves exhibit linearity and relatively symmetrical triangular shapes, indicating that this supercapacitor has good electrochemical reversibility. Figure 6e shows the CV curves of the MoO_3_//AC tested under different voltage windows after adding a certain proportion of EG to the electrolyte. When the voltage window is 2.2 V, certain polarization occurs and small bubbles appear on the electrode plates, suggesting that the voltage window is too large at this time. Therefore, the voltage window is determined to be 2.1 V. It can be seen that adding a certain proportion of EG to the electrolyte has successfully broadened the voltage window from 0–1.7 V to 0–2.1 V, achieving the goal of widening the voltage. Figure 6f presents the CV curves of two electrolytes, Na_2_SO_4_−EG and Na_2_SO_4_, at a scanning rate of 10 mV s^−1^ under a voltage window of 0–1.7 V. It is evident that the CV area of Na_2_SO_4_−EG is larger, indicating that it has more abundant electroactive sites, higher electronic conductivity, and more accessible electrolyte ions, which leads to a higher specific capacitance.

**Figure 7:**
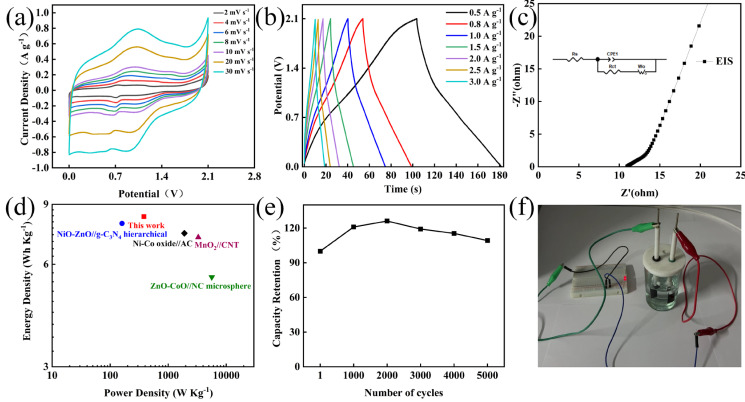
Electrochemical performance of MoO_3_//AC supercapacitors: (**a**) CV plot at 0−2.1 V; (**b**) GCD diagram at 0−2.1 V; (**c**) EIS diagram at 0−2.1 V; (**d**) Ragone plot (energy densities vs. power density); (**e**) cycle life diagram of supercapacitors (current density of 2 A g^−1^); (**f**) schematic diagram of supercapacitor device illuminating LED light.

Figure 7 shows the electrochemical performance tests of the MoO_3_//AC asymmetric supercapacitor assembled with the Na_2_SO_4_−EG electrolyte. Specifically, Figure 7a presents the CV curves at different scanning rates, demonstrating typical redox behaviors. Moreover, all the CV curves exhibit similar shapes. With the increase in the scanning rate, the positions of the oxidation peak and the reduction peak shift slightly towards the positive and negative potentials, respectively; this is mainly related to the polarization of the electrodes. Figure 7b shows the GCD curves of MoO_3_//AC. The approximately symmetrical GCD curves possess good coulombic efficiency and electrochemical reversibility, indicating that it has an excellent rate performance, ideal capacitive characteristics, and remarkable electrochemical reversibility [32]. The EIS test of the MoO_3_//AC device is shown in Figure 7c, where the inset is the simulated equivalent circuit diagram. The Rs and Rct values obtained through the fitting calculations are 2.6 Ω and 0.246 Ω, respectively. The above test results indicate that the MoO_3_//AC asymmetric device has relatively good capacitive characteristics. Figure 7d shows a Ragone plot comparing the electrochemical performance of supercapacitors with different electrodes. The supercapacitor can achieve an energy density of 8.28 Wh Kg^−1^ at a power density of 382.15 W Kg^−1^, which is superior to the previously reported hierarchical supercapacitor NiO ZnO//g−C_3_N_4_ (160 W Kg^−1^, 7.91 Wh Kg^−1^ [33]), microsphere ZnO−CoO//NC (5634.5 W Kg^−1^, 5.5 Wh Kg^−1^ [34]), MnO_2_//CNT(3300 W Kg^−1^, 7.2 Wh Kg^−1^ [35]), and Ni−Co oxide//AC (1902.9 W Kg^−1^, 7.4 Wh Kg^−1^ [36]). As shown in Figure 7e, the capacity retention rate of the MoO_3_//AC supercapacitor reached 109.2 % after being cycled 5,000 times at a current density of 2.0 A g^−1^. This is mainly due to the fact that the electrode material and the electrolyte ions make full contact during the cyclic charge–discharge process, thus improving the effective specific capacitance of the electrode. The prepared AC//MoO_3_ device can successfully light up the red LED lamp (Figure 7f) within a voltage window of 2.1 V, indicating that the MoO_3_ electrode material possesses excellent electrochemical properties and providing a new experimental basis for the preparation of supercapacitor electrode materials.

A table showing a comparison with previously published literature is provided in Table 2, showing that the aqueous MoO_3_//AC asymmetric supercapacitor assembled with the Na_2_SO_4_−EG electrolyte can retain more than 109.2% of its initial capacitance after 5000 cycles, because of the excellent mechanical stability and reduced volume expansion of MoO_3_ nanorods. Additionally, the MoO_3_//AC asymmetric supercapacitor displays the greatest working voltage (2.1 V), and this may be due to the strong coordination between EG molecules and Na+ ions; therefore, some H_2_O molecules are not in the Na^+^ solvation shell and do not participate in the decomposition process on the electrode surface, thus achieving a wide electrochemical window.

## 4. Conclusions

In this paper, the high aspect ratio of MoO_3_ nanorods provides more active sites for electrochemical reactions, and this unique morphology is achieved through a carefully optimized hydrothermal synthesis method (adjusting the hydrothermal temperature, hydrothermal time, and solution pH value) that precisely control the size and crystallinity of MoO_3_ nanorods. The advantages of high-performance MoO_3_ nanorods are their multivalence, numerous active sites, and high specific surface area, resulting in improved charge transfer reactions in the electrode/electrolyte and ion diffusion mobility. When the current density is 0.5 A g^−1^, the specific capacitance can reach 244.35 F g^−1^. Additionally, the assembled aqueous MoO_3_//AC asymmetric supercapacitor with Na_2_SO_4_−EG electrolyte can increase the working voltage from 1.7 V to 2.1 V, which is mainly attributable to the strong coordination between EG molecules and Na^+^ ions; therefore, some H_2_O molecules are not in the Na^+^ solvation shell and do not participate in the decomposition process on the electrode surface, thus achieving a wide electrochemical window (2.1 V). Meanwhile, a specific capacitance of 13.52 F g^−1^ can be achieved at a current density of 0.5 A g^−1^, an energy density of 8.28 Wh kg^−1^ and a power density of 382.15 W kg^−1^. The specific capacitance retention rate was 109.2 % after 5000 cycles, indicating that the prepared electrode materials possess a relatively high specific capacitance. In short, the assembled asymmetric supercapacitor successfully broadened the voltage window, and exhibited good electrochemical performance and cycling stability, providing new insights into regulating the wide voltage of supercapacitors from the perspective of electrolytes.

## Figures and Tables

**Table 1:** Comparison of BET of MoO_3_ prepared at different pH values.

Samples	PH = 0.36	PH = 0.58	PH = 0.84
BET surface area (m^2^ g^−1^)	7.9	7.6	7.5
BJH average pore size (nm)	65.0	48.7	40.0

**Table 2:** Performance comparison table of supercapacitors.

System	Voltage Window	Number of Cycles	Capacitance Retention Rate (%)	Ref.
MnO_2_ @GF//MoO_3_	0.85–1.55 V	300	81%	[37]
I_2_-NP-CP@Nafion//MoO_3_	0–1.8 V	500	93.2%	[8]
Cu_0.82_Co_0.18_HCF//h-MoO_3_	0–1.6 V	10,000	83%	[38]
YP50//MoO_3_	0–1.4 V	1500	84%	[39]
Graphene composite//MoO_3_−MoS_2_	−0.8–0 V	500	50.3%	[40]
AC//MoO_3_	0–2.1 V	5000	1092%	This work

## Data Availability

Data available upon request.

## References

[1] Chen J.L., Xu Z.F., Zhu H.L., Liu R., Song X.J., Song Q., Wu J., Zhang C., Ding L., Dong J. (2020). An ultrafast supercapacitor built by Co_3_O_4_ with tertiary hierarchical architecture. Vacuum.

[2] Guo R., Dang L.Q., Liu Z.H., Lei Z.B. (2020). Incorporation of electroactive NiCo_2_S_4_ and Fe_2_O_3_ into graphene aerogel for high-energy asymmetric supercapacitor. Colloid. Surf. A Physicochem. Eng. Asp..

[3] Libich J., Máca J., Vondrák J., Ech O., Sedlaíková M. (2018). Supercapacitors: Properties and applications. J. Energy Storage.

[4] Zhang M., Yang D.Y., Li J.T. (2020). Supercapacitor performances of MnO_2_ and MnO_2_/reduced graphene oxide prepared with various electrodeposition time. Vacuum.

[5] Mao X.L., Liu H., Xu J.H., Li M.W., Yang W.Y. (2024). Surface reconstruction of Co_3_O_4_/rGO heterointerface enabling high-performance asymmetric supercapacitors. J. Energy Storage.

[6] Pal S., Kumar Chattopadhyay K. (2018). Fabrication of Molybdenum Trioxide Nanobelts as High Performance Supercapacitor. Mater. Today Proc..

[7] Wang L.C., Gao L., Wang J., Shen Y. (2019). MoO_3_ nanobelts for high-performance asymmetric supercapacitor. J. Mater. Sci..

[8] Si H., Han C., Cui Y., Sang S., Liu K., Liu H., Wu Q. (2021). The electrochemical properties of iodine cathode in a novel rechargeable hydrogen ion supercapattery system with molybdenum trioxide as anode. Electrochim. Acta.

[9] Lahan H., Das S.K. (2019). Reversible Al^3+^ ion insertion into tungsten trioxide (WO_3_) for aqueous aluminum-ion batteries. Dalton Trans..

[10] Huang S., Li Z., Li P., Du X., Ma M., Liang Z., Su Y., Xiong L. (2023). Ultrahigh-voltage aqueous electrolyte for wide-temperature supercapacitors. J. Mater. Chem..

[11] D’Alessandro A., Bellani S., Gamberini A., Mastronardi V., Zappia M.I., Abruzzese M., Thorat S., Calcagno E., Bonaccorso F. (2024). Water-based supercapacitors with amino acid electrolytes: A green perspective for capacitance enhancement. Batter. Supercaps.

[12] Wang R.Y., Shi M.J., Li L.Y., Zhao Y., Zhao L.P., Yan C. (2023). In-situ investigation and application of cyano-substituted organic electrode for rechargeable aqueous Na-ion batteries. Chem. Eng. J..

[13] Liu S.Q., Klukas R., Porada T., Furda K., Fernandez A.M., Balducci A. (2022). Potassium formate-based electrolytes for high performance aqueous electrochemical capacitors. J. Power Sources.

[14] Nandagudi A., Nagarajarao S.H., Santosh M.S., Basavaraja B.M., Malode S.J., Mascarenhas R.J., Shetti N.P. (2022). Hydrothermal synthesis of transition metal oxides, transition metal oxide/carbonaceous material nanocomposites for supercapacitor applications. Mater. Today Sustain..

[15] Duan H., Zhao Z., Lu J., Hu W., Pang H. (2021). When Conductive MOFs Meet MnO_2_: High Electrochemical Energy Storage Performance in an Aqueous Asymmetric Supercapacitor. ACS Appl. Mater. Intefaces.

[16] Wang T.Y., Liu J., Ma Y.X., Han S., Gu C.D., Lian J.S. (2021). P-N heterojunction NiO/ZnO electrode with high electrochemical performance for supercapacitor applications. Electrochim. Acta.

[17] Cheng S.T., Zhang Y.X., Liu Y.P., Sun Z.H., Cui P., Zhang J.L., Hua X., Su Q., Fu J., Xie E. (2021). Energizing Fe_2_O_3_-based supercapacitors with tunable surface pseudocapacitance via physical spatial-confining strategy. Chem. Eng. J..

[18] Sun L., Liu Y., Yan M., Yang Q.J., Liu X.Y., Shi W.D. (2022). Lewis acid etched Ni_x_Co_1-x_Se_2_ derived from ZIF-L on CoO nanowires for hybrid-supercapacitors. Chem. Eng. J..

[19] De Castro I.A., Datta R.S., Ou J.Z., Castellanos-Gomez A., Sriram S., Daeneke T., Kalantar-zadeh K. (2017). Molybdenumoxides-from fundamentals to functionality. Adv. Mater..

[20] Hu X.L., Zhang W., Liu X.X., Mei Y.N., Huang Y. (2015). Nanostructured Mo-based electrode materials for electrochemical energy storage. Chem. Soc. Rev..

[21] Liu H., Liu X., Wang S.L., Liu H.K., Li L. (2020). Transition metal based battery-type electrodes in hybrid supercapacitors: A review. ESM.

[22] Pan Z.H., Yang C.H., Li Y., Hu X., Ji X.H. (2022). Rational design of A-CNTs/K_x_MnO_2_ and Ti_3_C_2_T_x_/MoO_3_ free-standing hybrid films for flexible asymmetric supercapacitor. Chem. Eng. J..

[23] Huang Z.Y., Zhang Z., Qi X., Ren X.H., Xu G.H., Wan P.B., Sun X.M., Zhang H. (2016). Wall-like hierarchical metal oxide nanosheet arrays grown on carbon cloth for excellent supercapacitor electrodes. Nanoscale.

[24] Zhang S.W., Yin B.S., Liu C., Wang Z.B., Gu D.M. (2017). Self-assembling hierarchical NiCo_2_O_4_/MnO_2_ nanosheets and MoO_3_/PPy core-shell heterostructured nanobelts for supercapacitor. Chem. Eng. J..

[25] Noby S.Z., Mohanty A., Zirak P., Ramadoss A., Schmidt-Mende L. (2022). Ramadoss, Hierarchical carbon coated vertically aligned α-MoO_3_ nanoblades anode materials for supercapacitor application. J. Alloys Compd..

[26] Yu C.Y., Xu H., Gong Y.J., Chen R.Y., Hui Z.Y., Zhao X., Sun Y., Chen Q., Zhou J., Ji W. (2021). The Jahn-Teller Effect for Amorphization of Molybdenum Trioxide towards High-Performance Fiber Supercapacitor. Research.

[27] Mao X.L., He X., Yang W.Y., Liu H., Zhou Y.J., Xu J.H., Yang Y. (2019). Hierarchical holey Co_9_S_8_@S-rGO hybrid electrodes for high-performance asymmetric supercapacitors. Electrochim. Acta.

[28] Hu H.M., Deng C.H., Xu J.C., Zhang K.H., Sun M. (2015). Metastable *h*-MoO_3_ and stable α-MoO_3_ microstructures: Controllable synthesis, growth mechanism and their enhanced photocatalytic activity. J. Exp. Nanosci..

[29] Yang J., Xiao X., Chen P., Zhu K., Cheng K., Ye K., Wang G., Cao D., Yan J. (2019). Creating oxygen-vacancies in MoO_3-x_ nanobelts toward high volumetric energy-density asymmetric supercapacitors with long lifespan. Nano Energy.

[30] Xu L.M., Zhou W.Q., Chao S.X., Liang Y.M., Zhao X.Q., Liu C.C., Xu J. (2022). Advanced Oxygen-Vacancy Ce-Doped MoO_3_ Ultrathin Nanoflakes Anode Materials Used as Asymmetric Supercapacitors with Ultrahigh Energy Density. Adv. Energy Mater..

[31] Saraf M., Shuck C.E., Norouzi N., Matthews K., Inman A., Zhang T., Pomerantseva E., Gogotsi Y. (2023). Free-Standing α-MoO_3_/Ti_3_C_2_ MXene Hybrid Electrode in Water-in-Salt Electrolytes. Energy Environ. Mater..

[32] Mao X., Xu J., He X., Yang W., Yang Y., Xu L., Zhao Y., Zhou Y. (2018). All-solid-state flexible microsupercapacitors based on reduced graphene oxide/multi-walled carbon nanotube composite electrodes. Applied Surface Science. Appl. Surf. Sci..

[33] Chen X., Wang X., Liu F., Song X., Cui H. (2020). Fabrication of NiO–ZnO-modified g C_3_N_4_ hierarchical composites for high-performance supercapacitors. Vacuum.

[34] Yao D., Wang F.L., Lei W., Hua Y., Xia X.F., Liu J.P., Hao Q. (2020). Oxygen vacancies boosting ultra-stability of mesoporous ZnO-CoO@N-doped carbon microspheres for asymmetric supercapacitors. Sci. China-Mater..

[35] Ko W.Y., Chen Y.F., Lu K.M., Lin K.J. (2016). Porous honeycomb structures formed from interconnected MnO_2_ sheets on CNT-coated substrates for flexible all-solid-state supercapacitors. Sci. Rep..

[36] Tang C., Tang Z., Gong H. (2012). Hierarchically Porous Ni-Co Oxide for High Reversibility Asymmetric Full-Cell Supercapacitors. J. Electrochem. Soc..

[37] Yan L., Huang J.H., Guo Z.W., Dong X.L., Wang Z., Wang Y.G. (2020). Solid-State Proton Battery Operated at Ultralow Temperature. ACS Energy Lett..

[38] Xu T.Z., Xu Z.M., Yao T.Y., Zhang M., Chen D., Zhang X., Shen L. (2023). Discovery offast and stableproton storage in bulk hexagonal molybdenum oxide. Nat. Commun..

[39] Zhu Y.L., Tan Y.T., Li H. (2023). MoO_3_ nanoplates preparation via self-sacrifice C_3_N_4_ for supercapacitors in an acid electrolyte. J. Energy Storage.

[40] Iamprasertkun P., Hirunpinyopas W., Tripathi A.M., Bissett M.A., Dryfe R.A.W. (2019). Electrochemical intercalation of MoO_3_-MoS_2_ composite electrodes: Charge storage mechanism of non-hydrated cations. Electrochim. Acta.

